# The role of physical activity in metabolic homeostasis before and after the onset of type 2 diabetes: an IMI DIRECT study

**DOI:** 10.1007/s00125-019-05083-6

**Published:** 2020-01-30

**Authors:** Robert W. Koivula, Naeimeh Atabaki-Pasdar, Giuseppe N. Giordano, Tom White, Jerzy Adamski, Jimmy D. Bell, Joline Beulens, Søren Brage, Søren Brunak, Federico De Masi, Emmanouil T. Dermitzakis, Ian M. Forgie, Gary Frost, Torben Hansen, Tue H. Hansen, Andrew Hattersley, Tarja Kokkola, Azra Kurbasic, Markku Laakso, Andrea Mari, Timothy J. McDonald, Oluf Pedersen, Femke Rutters, Jochen M. Schwenk, Harriet J. A. Teare, E. Louise Thomas, Ana Vinuela, Anubha Mahajan, Mark I. McCarthy, Hartmut Ruetten, Mark Walker, Ewan Pearson, Imre Pavo, Paul W. Franks, for the IMI DIRECT Consortium

**Affiliations:** 1 Department of Clinical Sciences, Lund University, Genetic and Molecular Epidemiology, CRC, Skåne University Hospital Malmö, Building 91, Level 12, Jan Waldenströms gata 35, SE-205 02 Malmö, Sweden; 2 Oxford Centre for Diabetes, Endocrinology and Metabolism, Radcliffe Department of Medicine, University of Oxford, Oxford, UK; 3 MRC Epidemiology Unit, University of Cambridge School of Clinical Medicine, Cambridge, UK; 4 Research Unit Molecular Endocrinology and Metabolism, Genome Analysis Center, Helmholtz Zentrum München, German Research Center for Environmental Health, Neuherberg, Germany; 5 Lehrstuhl für Experimentelle Genetik, Technische Universität München, Freising-Weihenstephan, Germany; 6 Department of Biochemistry, Yong Loo Lin School of Medicine, National University of Singapore, Singapore, Republic of Singapore; 7 Research Centre for Optimal Health, Department of Life Sciences, University of Westminister, London, UK; 8 Department of Epidemiology and Biostatistics, Amsterdam Public Health Research Institute, Amsterdam University Medical Centre, location VU University Medical Center, Amsterdam, the Netherlands; 9 Faculty of Health Sciences, University of Southern Denmark, Odense, Denmark; 10 The Novo Nordisk Foundation Center for Protein Research, University of Copenhagen, Copenhagen, Denmark; 11 Department of Bio and Health Informatics, Technical University of Denmark, Lyngby, Denmark; 12 Department of Genetic Medicine and Development, University of Geneva Medical School, Geneva, Switzerland; 13 Institute of Genetics and Genomics in Geneva (iGE3), University of Geneva, Geneva, Switzerland; 14 Swiss Institute of Bioinformatics, Geneva, Switzerland; 15 Population Health & Genomics, School of Medicine, University of Dundee, Ninewells Hospital, Dundee, UK; 16 Nutrition and Dietetics Research Group, Department of Medicine, Division of Diabetes, Endocrinology and Metabolism, Imperial College London, Hammersmith Campus, London, UK; 17 The Novo Nordisk Foundation Center for Basic Metabolic Research, Faculty of Health and Medical Science, University of Copenhagen, Copenhagen, Denmark; 18 NIHR Exeter Clinical Research Facility, University of Exeter Medical School, Exeter, UK; 19 Institute of Biomedical and Clinical Science, University of Exeter Medical School, Exeter, UK; 20 Department of Medicine, University of Eastern Finland and Kuopio University Hospital, Kuopio, Finland; 21 Institute of Neurosciences, National Research Council, Padova, Italy; 22 Affinity Proteomics, Science for Life Laboratory, KTH - Royal Institute of Technology, Stockholm, Sweden; 23 HeLEX, Nuffield Department of Population Health, University of Oxford, Old Road Campus, Headington, Oxford, UK; 24 Wellcome Centre for Human Genetics, University of Oxford, Oxford, UK; 25 NIHR Oxford Biomedical Research Centre, Churchill Hospital, Oxford, UK; 26 Present Address: Human Genetics, Genentech, South San Francisco, CA USA; 27 Sanofi-Aventis Deutschland GmbH, R&D, Frankfurt am Main, Germany; 28 Institute of Cellular Medicine (Diabetes), Newcastle University, Newcastle upon Tyne, UK; 29 Eli Lilly Regional Operations GmbH, Vienna, Austria; 30 Department of Nutrition, Harvard School of Public Health, Boston, MA USA; 31 Department of Public Health & Clinical Medicine, Section for Medicine, Umeå University Hospital, Umeå, Sweden

**Keywords:** Beta cell function, Ectopic fat, Glycaemic control, Insulin sensitivity, Physical activity, Prediabetes, Structural equation modelling, Type 2 diabetes

## Abstract

**Aims/hypothesis:**

It is well established that physical activity, abdominal ectopic fat and glycaemic regulation are related but the underlying structure of these relationships is unclear. The previously proposed twin-cycle hypothesis (TC) provides a mechanistic basis for impairment in glycaemic control through the interactions of substrate availability, substrate metabolism and abdominal ectopic fat accumulation. Here, we hypothesise that the effect of physical activity in glucose regulation is mediated by the twin-cycle. We aimed to examine this notion in the Innovative Medicines Initiative Diabetes Research on Patient Stratification (IMI DIRECT) Consortium cohorts comprised of participants with normal or impaired glucose regulation (cohort 1: *N* ≤ 920) or with recently diagnosed type 2 diabetes (cohort 2: *N* ≤ 435).

**Methods:**

We defined a structural equation model that describes the TC and fitted this within the IMI DIRECT dataset. A second model, twin-cycle plus physical activity (TC-PA), to assess the extent to which the effects of physical activity in glycaemic regulation are mediated by components in the twin-cycle, was also fitted. Beta cell function, insulin sensitivity and glycaemic control were modelled from frequently sampled 75 g OGTTs (fsOGTTs) and mixed-meal tolerance tests (MMTTs) in participants without and with diabetes, respectively. Abdominal fat distribution was assessed using MRI, and physical activity through wrist-worn triaxial accelerometry. Results are presented as standardised beta coefficients, SE and *p* values, respectively.

**Results:**

The TC and TC-PA models showed better fit than null models (TC: χ^2^ = 242, *p* = 0.004 and χ^2^ = 63, *p* = 0.001 in cohort 1 and 2, respectively; TC-PA: χ^2^ = 180, *p* = 0.041 and χ^2^ = 60, *p* = 0.008 in cohort 1 and 2, respectively). The association of physical activity with glycaemic control was primarily mediated by variables in the liver fat cycle.

**Conclusions/interpretation:**

These analyses partially support the mechanisms proposed in the twin-cycle model and highlight mechanistic pathways through which insulin sensitivity and liver fat mediate the association between physical activity and glycaemic control.

**Electronic supplementary material:**

The online version of this article (10.1007/s00125-019-05083-6) contains peer-reviewed but unedited supplementary material, which is available to authorised users.

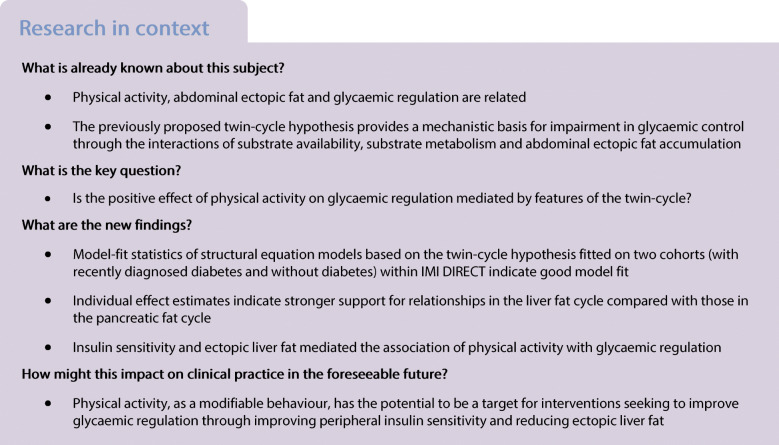



## Introduction

The global epidemics of type 2 diabetes and obesity [1, 2] follow in the wake of rapid urbanisation, reduced physical activity, and ageing populations [3]. Physical inactivity is strongly associated with peripheral insulin resistance, abdominal obesity and glucose dysregulation [4–7]. Physical inactivity may also predispose to non-alcoholic fatty liver disease (NAFLD) [8], which in turn may also adversely affect glucose homeostasis [9–11]. Indeed, recent Mendelian randomisation studies hint at bidirectional causal relationships between NAFLD and type 2 diabetes [12, 13]. Various mechanisms for these relationships have been proposed [14], with chronic positive energy balance considered a primordial modifiable risk factor [15, 16]. This notion is articulated through the twin-cycle model, whereby the first cycle describes liver fat accumulation leading to reduced suppression of hepatic gluconeogenesis, consequential elevations in both fasting glucose and insulin concentrations, and hepatic lipid production; the second cycle focuses on the pancreas, where elevated circulating lipids accumulate in the pancreas, impairing endogenous insulin secretion [15, 16]. However, the extent to which physical activity affects blood glucose homeostasis through mechanisms outlined in the twin-cycle model is unknown.

A better understanding of this would not only add to the physiological understanding of diabetes but might also help guide the design of clinical trials seeking to study the pathogenesis of type 2 diabetes. For example, as physical activity is a modifiable behaviour, it is a potential target for interventions seeking to modify processes involved in the relationships between NAFLD and type 2 diabetes. Furthermore, studies seeking to assess these relationships would benefit from understanding which factors are affected by physical activity and mediate the effect of physical activity in glycaemic control.

Multivariate structural analyses (such as structural equation modelling) can be a powerful way to address these putative effects but require accurate and concurrent assessments of glycaemic control, abdominal fat distribution and lifestyle variables in adequately sized cohorts, few of which currently exist. The Innovative Medicines Initiative Diabetes Research on Patient Stratification (IMI DIRECT) cohorts [17, 18] are well-suited for such analyses.

The purpose of this study was to test potential mechanisms mediating the effects of physical activity in glycaemic control before and after the onset of type 2 diabetes.

## Methods

### Study cohorts

These analyses were conducted in two parallel cross-sectional cohorts of European ancestry adults from northern Europe: the first cohort (cohort 1) comprised of participants with blood glucose concentrations within the normal glucose control or prediabetes (impaired HbA_1c_, fasting glucose or 2 h glucose according to ADA criteria [19]) brackets and the second cohort (cohort 2) comprised individuals with recently diagnosed type 2 diabetes (within 6–36 months of study enrolment). Participants underwent detailed physical examinations, including MRI scans and carbohydrate challenge tests, diet assessment and objective habitual physical activity assessment. Approval for the study protocol was obtained from each of the regional research ethics review boards separately and all participants provided written informed consent at enrolment. The research conformed to the ethical principles for medical research involving human participants outlined in the declaration of Helsinki.

The study rationale and design and core characteristics of the IMI DIRECT cohorts are reported in detail elsewhere [17, 18]. Below, we provide a summary and describe the methods most relevant for the present analyses.

Cohort 1 (prediabetes) was from a sampling frame of 24,196 participants nested within prospective cohorts from Denmark (Copenhagen), Finland (Kuopio), the Netherlands (Hoorn) and Sweden (Malmö); 2127 participants at varying risk of glycaemic deterioration were enrolled into the study. To determine the risk of rapid glycaemic deterioration, we used the DIRECT-DETECT algorithm [20]. For cohort 2 (diabetes), 789 participants were recruited from health registries and primary care practices in Denmark (Copenhagen), the UK (Dundee, Exeter, Newcastle), the Netherlands (Hoorn) and Sweden (Lund). As neither of the Swedish study centres undertook MRI scans, they were not included in the current analysis.

Of these participants, 920 (cohort 1) and 435 (cohort 2) had all the necessary variables for a complete case analysis of the twin-cycle hypothesis (TC) model and 725 (cohort 1) and 361 (cohort 2) had all the necessary variables for the complete case analyses fitting the twin-cycle plus physical activity (TC-PA) model. The following variables were included in these models: fasting plasma glucose, 2 h glucose, oral glucose insulin sensitivity (OGIS), liver fat, pancreatic fat, fasting insulin secretion rate, glucose sensitivity (insulin secretion per glucose), age, sex, centre, metformin use, total daily energy intake, total daily carbohydrate, protein and fat intake, and mean daily physical activity intensity. The characteristics of these subcohorts are shown in Table 1.

**Table 1:** Characteristics of cohort subset used in each model

Characteristic	Cohort 1 (no diabetes/prediabetes)		Cohort 2 (diabetes)	
	TC	TC-PA	TC	TC-PA
Male sex, %	83	83	57	60
Age, years	60.6 (6.3)	60.6 (6.3)	61.5 (8.3)	61.7 (8.4)
BMI, kg/m^2^	27.8 (3.6)	27.8 (3.7)	30.5 (4.8)	30.4 (4.6)
Fasting glucose, mmol/l	5.8 (0.5)	5.8 (0.5)	7 (1.5)	7 (1.4)
2 h glucose, mmol/l	6 (1.7)	6 (1.7)	8.6 (2.9)	8.5 (2.8)
Fasting triacylglycerol, mmol/l	1.4 (0.6)	1.4 (0.7)	1.5 (0.9)	1.5 (0.9)
Fasting insulin, pmol/l	73 (49)	75 (51)	105 (69)	105 (68)
Fasting insulin secretion, pmol min^−1^ m^−2^	105 (40)	106 (41)	134 (49)	136 (50)
Glucose sensitivity, pmol min^−1^ m^−2^ mmol l^−1^	107 (50)	107 (50)	85 (54)	89 (56)
Insulin sensitivity, 2 h OGIS, ml min^−1^ m^−2^	374 (56)	374 (56)	302 (71)	302 (70)
Liver fat, %	5 (4.7)	5 (4.7)	8.6 (7.2)	8.9 (7.4)
Pancreatic fat, %	13.5 (9)	13.6 (9.1)	11.2 (7.2)	11.7 (6.9)

Values are mean (SD), except for male sex, which is % of subcohort

### Measures

Fasting glucose was assessed from venous plasma samples drawn in the morning following an overnight fast. Frequently sampled 75 g oral glucose tolerance tests (fsOGTTs) and mixed-meal tolerance tests (MMTTs) were carried out in cohort 1 and 2, respectively. Mixed meals (250 ml Fortisip liquid drink [18.4 g carbohydrate per 100 ml]) rather than 75 g oral glucose loads were used in cohort 2 to minimise the risk of severe hyperglycaemia as participants had type 2 diabetes. The 2 h glucose, OGIS, fasting insulin secretion rate and glucose sensitivity (dose–response slope of insulin secretion in response to glucose) were calculated from the fsOGTT and MMTT data, as described elsewhere [21, 22]. Liver and pancreatic fat were measured by MRI and quantified using a multi-echo technique described in detail elsewhere [17, 18, 23, 24]. Briefly, prone 1.5 T to 3 T images (depending on availability at each study centre) were acquired. T1-weighted images were obtained for the abdominal region (between the diaphragm and acetabulum) with maximum field of view and 10 mm slice thickness with a 10 mm slice gap. A three-dimensional scan using 50–80 images at slice thicknesses of 1.2–2 mm (depending on equipment) was acquired to image the pancreas. Further axial single-slice multi-echo images were acquired of the liver and pancreas (10 mm slice thickness). Whole-organ pancreatic and liver fat estimates were then inferred from these images, where experienced radiographers manually determined organ boundaries. Physical activity was objectively assessed using triaxial accelerometry (ActiGraph GT3X+, ActiGraph, Pensacola, FL, USA) on the non-dominant wrist over 10 days. Physical activity intensity was characterised by calculating the mean high-pass filtered vector magnitude (hpfVM) of the triaxial acceleration signal [25, 26]; the mean of this was used to describe overall physical activity level. Non-wear was inferred as a vector magnitude SD of less than 4 m*g* for a consecutive period greater than 60 min. To account for bias introduced by removal of non-wear time in combination with differential diurnal non-wear patterns between individuals, adjustments were made for diurnal rhythm [27]. Dietary intake was assessed using a validated multi-pass food habit questionnaire and 24 h diet record, as previously described [17, 18].

### Statistical analysis

All continuous variables were standardised by rank-normal transformed (mean 0, SD 1) by sex (and by lifestyle vs metformin + lifestyle in cohort 2). Adjustment for putative confounders was done by two-step residual regression where, in the first step, residuals were extracted from general linear models undertaken on the transformed continuous variables or binary categorical variables, and these residuals were used in subsequent models as either outcome or predictor variables. Regression models for residual extraction co-varied for age, study centre, total daily energy intake and total daily intake of dietary carbohydrates, fats and proteins. Pearson correlation coefficients were generated and plotted in a matrix to illustrate simple pairwise relationships between all model variables.

### Structural equation modelling

**Fig. 1:**
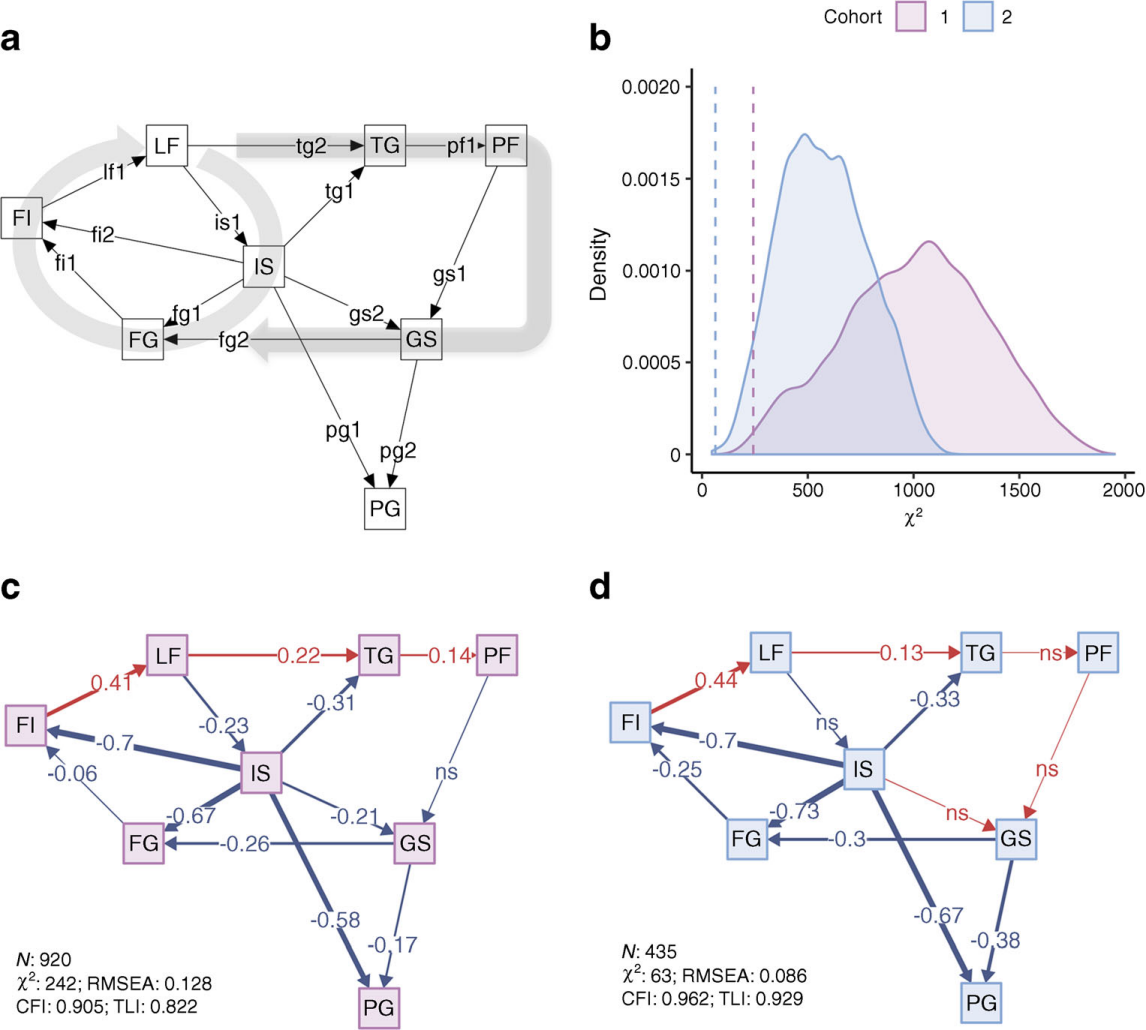
TC structural equation model definition diagram, fit estimates and effect estimate diagrams from a hypothesised model for the role of physical activity and liver fat in glycaemic control. (**a**) Model definitions, with squares representing manifest nodes and arrows indicating regression coefficients pointing towards an outcome of a respective regression. (**b**) Model fit; density plot of model fit χ^2^ from variable-randomised comparable structural equation models applied on respective dataset (10,000 iterations). Dashed vertical lines indicate TC model χ^2^, solid lines and shaded areas indicate χ^2^ of all null iterations. (**c**, **d**) Effect estimate diagrams of the defined model applied on cohort 1 (no diabetes/prediabetes, **c**) and cohort 2 (type 2 diabetes, **d**), where the arrow thickness is weighted by effect estimate magnitude, and colours red and blue indicate positive and negative estimates, respectively. All continuous variables are normally transformed and adjusted for age, sex, metformin treatment (cohort 2), study centre, total energy intake, and carbohydrate, fat and protein intake. See Text box for node and edge abbreviations

**Fig. 2:**
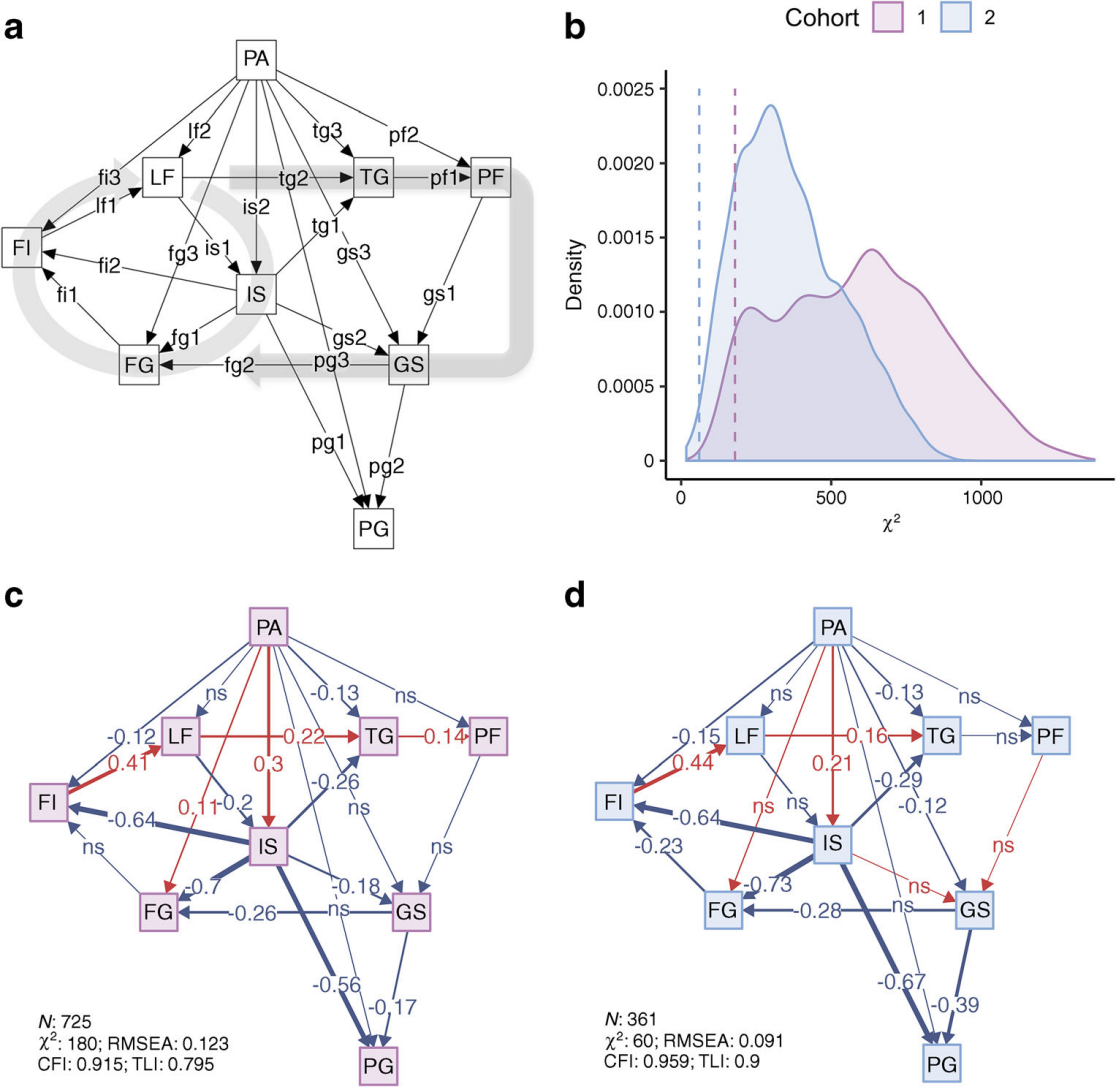
TC-PA structural equation model definition diagram, fit estimates and effect estimate diagrams from a hypothesised model for the role of physical activity and liver fat in glycaemic control. (**a**) Model definitions, with squares representing manifest nodes and arrows indicating regression coefficients pointing towards an outcome of a respective regression. (**b**) Model fit; density plot of model fit χ^2^ from variable-randomised comparable structural equation models applied on respective dataset (10,000 iterations). Dashed vertical lines indicate TC-PA model χ^2^, solid lines and shaded areas indicate χ^2^ of all null iterations. (**c**, **d**) Effect estimate diagrams of the defined model applied on cohort 1 (no diabetes/prediabetes, **c**) and cohort 2 (type 2 diabetes, **d**), where the arrow thickness is weighted by effect estimate magnitude, and colours red and blue indicate positive and negative estimates, respectively. All continuous variables are normally transformed and adjusted for: age, sex, metformin treatment (cohort 2), study centre, total energy intake, and carbohydrate, fat and protein intake. See Text box for node and edge abbreviations


We used structural equation modelling to test the overall model fit and relationships between sets of variables within the hypothesised twin-cycle. Structural equation modelling is a multivariate statistical method that can be thought of as a combination of regression analysis, factor analysis and pathway analysis. In a structural equation model a structure (a pathway network) of relationships between variables can be defined according to a prespecified hypothesis (such as the twin-cycle). Based on the observed covariance between the variables in the model, the fit of the defined model can then be tested. In addition to this, pathway (mediation) effects within the defined model can also be tested. We defined models using measured variables only (manifest nodes) and fitted under a maximum likelihood framework using covariance matrices. The model definition (see Figs 1a, 2a) reflects the hypothesised twin-cycle model, proposed elsewhere [15, 16]. Here, relationships (edge estimates) between variables are adjusted for putative confounders (through two-step residual regression, described above, hence fitted on covariance matrices of the extracted residuals). Direct (non-mediated) edge estimates also account for covariance of the other edges pointing to the same outcome. In other words, edges (see arrows in Figs 1, 2) in the model represent regression coefficients, which co-vary with edges from other variables pointing to the same outcome node (see Text box for node and edge abbreviations). Pathway (indirect) effects were estimated for mediated associations of physical activity with glycaemic control using the coefficient product method [28], where mediation is defined using the approach described by Baron and Kenny [29]. Pathways were tested where statistically significant direct associations (individual, non-mediated, edge estimates) along the whole pathway were also observed. Relative model fit was assessed using the comparative fit index (CFI) and the Tucker–Lewis index (TLI), with values ranging from 0 (no fit) to 1 (perfect fit) [30]; a model with a ‘good’ fit typically requires both indices to exceed 0.95 [31, 32]. Absolute fit was assessed using root mean square error of approximation (RMSEA). This ranges from 0 to 1, with 0 indicating a perfect fit [30]. A poorly fitting model is typically defined by RMSEA >0.06 [33, 34]. CFI, TLI and RMSEA were not used to formally determine adequacy of fit, as their use in this context is controversial and there is limited consensus on appropriate cut-off values because each index is affected differently by degrees of freedom, model complexity and sample size; it is, however, standard practice to report these along with the χ^2^. To overcome this, we formally tested model fit by comparing the χ^2^ of the tested model with χ^2^ values obtained from variable-randomised null models with identical structures (in other words, the variables were randomly assigned to other nodes in the same structural equation model definition) and applied to the respective covariance matrix used for the tested model. This process was iterated 10,000 times. To determine whether the tested model χ^2^ values were lower (fitted better) than mean χ^2^ values, one-sample *t* tests were used. We also calculated the empirical probability of the χ^2^ value from the null model being lower than the χ^2^ value from the tested model by expressing the tested model χ^2^ value as a quantile within the iterated χ^2^ values.

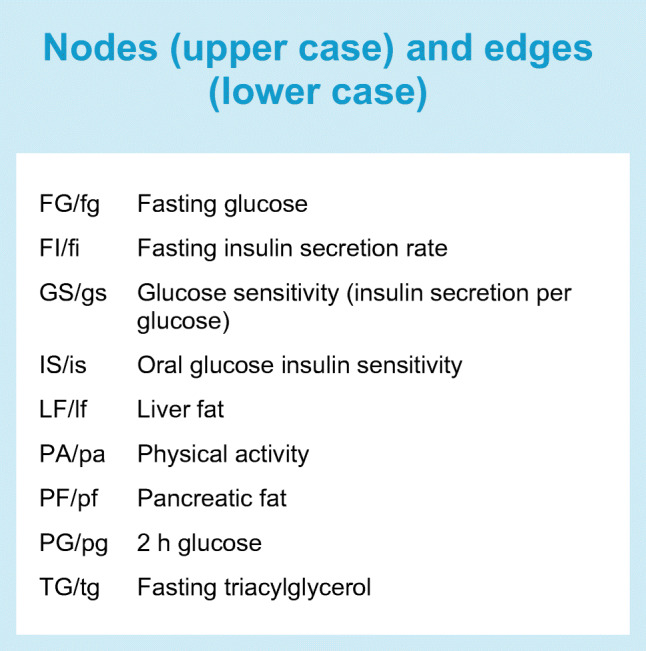


Multiple testing adjustments were not undertaken, as this analysis sought to validate a single previously hypothesised model where direct effect estimates were nested within this single model, reflecting a single overarching hypothesis. Moreover, where results are consistent across the two subcohorts used here, one might regard this as replication. However, the absence of replication may reflect real differences in diseased and non-diseased states, as opposed to providing evidence of type 1 error.

All statistics were computed using *R* version 3.5.0 [35]. Structural equation models were fitted using lavaan version 0.6-5 [36]. Models were plotted using semPlot version 1.1.2 (CRAN repository or https://github.com/SachaEpskamp/semPlot, accessed 20 August 2019). The IMI DIRECT data release version used for these analyses was ‘direct_29-03-2019’.

## Results

### Pairwise correlations

Statistically significant pairwise correlations between most of the index metabolic outcomes and physical activity were observed, thereby justifying the main structural equation model analysis to test the underlying structure of these correlations. An overview of the pairwise correlations is presented in electronic supplementary material (ESM) Fig. 1. Of note, in both cohorts, indices of reduced insulin resistance such as the increase of OGIS and the suppression of fasting insulin secretion rate correlated strongly with physical activity. However, pairwise associations between pancreatic fat and glucose sensitivity or fasting glucose were not observed, despite the presence of associations between pancreatic fat and a number of other index metabolic variables in cohort 1.

### Structural equation model

Structural equation model results are shown in Figs 1 and 2 for TC and TC-PA models, respectively. Effect estimates are presented in Table 2 for direct effects for TC and TC-PA models. Indirect (mediation/pathway) effect estimates of physical activity in fasting glucose and 2 h glucose variation from the TC-PA model are presented in Table 3. In the model definition diagrams in Figs 1a and 2a, we illustrate which direct effects (edges, depicted as arrows) were modelled within the structural equation model. We also illustrate the underlying liver cycle and pancreatic cycle within the TC to orientate the reader to the original hypothesis [16]. Below, we first describe the overall model fit and key direct (individual edges) effect estimates between physical activity and the index metabolic outcomes within the model. We then describe indirect (pathway) effect estimates between physical activity and glycaemic regulation mediated by twin-cycle variables.

**Table 2:** Individual edge effect estimates for the TC and TC-PA structural equation models

Outcome node/parent node (edge)	Cohort 1 (no diabetes/prediabetes)			Cohort 2 (diabetes)		
	β	β SE	*p* value	β	β SE	*p* value
TC model						
OGIS/						
LF (is1)	−0.23	0.04	<0.001	−0.11	0.06	0.09
LF/						
FI (lf1)	0.41	0.04	<0.001	0.44	0.05	<0.001
FI/						
FG (fi1)	−0.06	0.03	0.05	−0.25	0.06	<0.001
IS (fi2)	−0.70	0.03	<0.001	−0.70	0.06	<0.001
FG/						
IS (fg1)	−0.67	0.03	<0.001	−0.73	0.03	<0.001
GS (fg2)	−0.26	0.03	<0.001	−0.31	0.03	<0.001
TG/						
IS (tg1)	−0.31	0.03	<0.001	−0.34	0.05	<0.001
LF (tg2)	0.22	0.03	<0.001	0.13	0.05	0.006
PF/						
TG (pf1)	0.14	0.03	<0.001	0.01	0.05	0.886
GS/						
PF (gs1)	−0.05	0.04	0.185	<0.01	0.05	0.967
IS (gs2)	−0.21	0.03	<0.001	0.02	0.05	0.63
PG/						
IS (pg1)	−0.58	0.03	<0.001	−0.67	0.03	<0.001
GS (pg2)	−0.17	0.03	<0.001	−0.38	0.03	<0.001
TC-PA model						
IS/						
PA (is2)	0.30	0.04	<0.001	0.21	0.05	<0.001
LF (is1)	−0.20	0.04	<0.001	−0.12	0.07	0.065
LF/						
PA (lf2)	−0.04	0.04	0.234	−0.04	0.05	0.447
FI (lf1)	0.41	0.04	<0.001	0.44	0.05	<0.001
FI/						
PA (fi3)	−0.12	0.03	<0.001	−0.16	0.05	0.001
FG (fi1)	−0.04	0.03	0.265	−0.23	0.07	<0.001
IS (fi2)	−0.64	0.04	<0.001	−0.64	0.07	<0.001
FG/						
PA (fg3)	0.11	0.03	0.001	0.06	0.03	0.061
IS (fg1)	−0.70	0.03	<0.001	−0.73	0.03	<0.001
GS (fg2)	−0.26	0.03	<0.001	−0.28	0.03	<0.001
TG/						
PA (tg3)	−0.13	0.04	<0.001	−0.13	0.05	0.012
OGIS (tg1)	−0.26	0.04	<0.001	−0.30	0.05	<0.001
LF (tg2)	0.22	0.04	<0.001	0.16	0.05	0.002
PF/						
PA (pf2)	−0.06	0.03	0.067	−0.01	0.06	0.809
TG (pf1)	0.14	0.03	<0.001	−0.01	0.05	0.819
GS/						
PA (gs3)	−0.05	0.04	0.212	−0.12	0.06	0.032
PF (gs1)	−0.05	0.05	0.314	0.01	0.05	0.835
IS (gs2)	−0.18	0.04	<0.001	0.02	0.05	0.696
PG/						
PA (pg3)	−0.04	0.03	0.194	−0.05	0.04	0.162
IS (pg1)	−0.56	0.03	<0.001	−0.67	0.03	<0.001
GS (pg2)	−0.17	0.03	<0.001	−0.39	0.03	<0.001

All continuous variables were normally transformed and adjusted for age, sex, metformin treatment (cohort 2), study centre, total energy intake and carbohydrate, fat and protein intake

See Text box for node and edge abbreviations

**Table 3:** Pathway (mediation) effect estimates for the association of physical activity with glycaemic control within the TC-PA model (see Fig. 2)

		Cohort 1 (no diabetes/prediabetes)			Cohort 2 (diabetes)		
Outcome node	Edge path	β	β SE	*p* value	β	β SE	*p* value
FG	PA→IS→FG	−0.212	0.026	<0.001	−0.153	0.039	<0.001
PG	PA→IS→PG	−0.171	0.022	<0.001	−0.140	0.036	<0.001
FG	PA→IS→GS→FG	0.015	0.004	<0.001			
PG	PA→IS→GS→PG	0.009	0.003	0.001			
FG	PA→FI→LF→IS→FG	−0.007	0.002	0.002			
PG	PA→FI→LF→IS→PG	−0.006	0.002	0.002			
FG	PA→GS→FG				0.033	0.016	0.037
PG	PA→GS→PG				0.045	0.022	0.04

All continuous variables are normally transformed and adjusted for age, sex, metformin treatment (cohort 2), study centre, total energy intake and carbohydrate, fat and protein intake

See Text box for node abbreviations

#### Model fit

The TC model showed better fit than the mean fit of the respective null model in cohort 1 (χ^2^ = 242 vs 1005, *p* < 5 × 10^−10^) and cohort 2 (χ^2^ = 63 vs 587, *p* < 5 × 10^−10^) (see Figs 1b, 2b.). The fit from a randomised null model (10,000 iterations) was unlikely to be better than the TC model in cohort 1 (empirical *p* = 0.004) and cohort 2 (empirical *p* = 0.001). The TC-PA model also showed better fit than the mean fit of the respective null model in cohort 1 (χ^2^ = 180 vs 605, *p* < 5 × 10^−10^) and cohort 2 (χ^2^ = 60 vs 369, *p* < 5 × 10^−10^). The fit from a randomised null model was unlikely to be better than the TC model in cohort 1 (empirical *p* = 0.041) and cohort 2 (empirical *p* = 0.008).

#### Direct (non-mediated) effects

Most direct effects estimates in the TC model were statistically significant and were in a direction consistent with this hypothesis in both cohorts (Table 2). One notable exception was the relationship between pancreatic fat and glucose sensitivity, which was not statistically significant in either cohort. Physical activity bore a direct positive association with insulin sensitivity in both cohorts. An inverse direct association between physical activity and fasting insulin secretion was also observed in both cohorts. Physical activity was not directly associated with liver fat or 2 h glucose in either cohort. An inverse direct association between physical activity and fasting triacylglycerol was observed in both cohorts. Physical activity was inversely associated with glucose sensitivity in cohort 2 only.

#### Indirect (pathway/mediation) effects

The association of physical activity with glycaemic control was primarily mediated by variables in the liver fat cycle. Physical activity was associated with fasting glucose through pathway PA→IS↑→FG↓ in both cohorts and through pathways PA→FI↓→LF↓→IS↑→FG↓, PA→IS↑→GS↓→FG↑, in cohort 1. Consistently, physical activity was associated with 2 h glucose through pathway PA→IS↑→PG↓ in both cohorts and through pathway PA→FI↓→LF↓→IS↑→PG↓, PA→IS↑→GS↓→PG↑ in cohort 1. However, physical activity was associated with both increased fasting glucose and 2 h glucose through pathways PA→GS↓→FG↑ and PA→GS↓→PG↑ in cohort 2 only. See Table 3 for effect estimates and pathway details and Text box for node abbreviations.

## Discussion

Using structural equations to fit models describing the TC [15, 16], we found that in adults with prediabetes and type 2 diabetes overall physical activity volume is associated with multiple metabolic and abdominal ectopic fat features. We also demonstrate that reduced whole-body insulin sensitivity and fasting insulin secretion rate mediate the effects of physical activity in glucose and liver fat homeostasis. This is to our knowledge the first detailed pathway analysis of physical activity and glycaemic regulation.

Our findings partially support the validity of the TC proposed by Taylor [15, 16]. However, our data do not support the hypothesised effect of pancreatic fat in beta cell function, despite finding that pancreatic fat is associated with numerous other metabolic features; notably, these associations were observed both in conventional pairwise analyses (ESM Fig. 1) and within structural equation models (Figs 1, 2 and Table 2). This is important, as these positive results mitigate the possibility that the absence of associations between pancreatic fat and beta cell function is due to measurement error. A key benefit of the approach used here is that the magnitude of the effects of physical activity on glycaemic control is quantified (Table 3), so is likely to prove useful for those planning interventions related to this topic.

The direct effects observed here between liver fat accumulation and reduced insulin sensitivity support a central role for the liver in mediating the effects of physical activity in glycaemic control (see Fig. 1 and Table 2). Specifically, the PA→IS↑, IS→FI↓, FI→LF↑, IS→FG↓ and IS→PG↓ associations were statistically robust within and between cohorts. Indeed, pathways PA→IS↑→FG↓ and PA→IS↑→PG↓ are consistent in direction and magnitude in both cohorts, providing reassurance that these findings are not false positives, and suggesting that physical activity exerts effects on glucose homeostasis via insulin sensitivity in a similar way before and after the onset of type 2 diabetes. The pathways PA→FI↓→LF↓→IS↓→FG↓ and PA→FI↓→LF↓→IS↑→PG↓ were only tested in cohort 1, as the direct effect estimate LF→IS was not statistically significant in cohort 2.

A core feature of the TC relates to the role of islet triacylglycerol content in beta cell lipotoxicity, leading to diminished beta cell function [16]. As beta cells are estimated to account for only a small fraction of total pancreatic volume (<5% [37]), MRI imaging of the pancreas may be too insensitive to specifically quantify beta cell triacylglycerol content, with the signal instead being driven by whole pancreas fat content [38]. Furthermore, the serrations and involutions that characterise some pancreases [39] make the accurate assessment of ectopic fat near the boundary of the organ challenging, even using whole-organ MRI techniques and experienced radiographers identifying organ boundaries, as was the case here. The shape of the pancreas may also influence (or be correlated with factors that influence) diabetes remission following very-low-energy diets, such that improvements in early insulin secretion are greater in individuals with regularly rather than irregularly shaped pancreases [40]. However, there is a clear possibility that if pancreas shape and pancreatic fat measurement error correlate, the apparent relationship of pancreas fat and beta cell function may not be causal. Notwithstanding the difficulty in assessing pancreatic fat, we do observe pairwise correlations between pancreatic fat and the other metabolic outcomes in cohort 1 (see ESM Fig. 1), consistent with observations made by Tushuizen et al [41] who noted a relationship between pancreatic fat and beta cell function in prediabetes but not type 2 diabetes. Thus, pancreatic fat may have a significant role in glucose homeostasis, though we could not determine in our analyses if this is mediated through beta cell function.

As anticipated, we also observed differences in the association of insulin sensitivity with beta cell function between the prediabetes and the diabetes cohorts. In the prediabetes cohort, beta cell function and insulin sensitivity were related; this was not the case in the diabetes cohort, probably because the capacity to compensate for peripheral insulin resistance by secreting more insulin is greater in prediabetes than once diabetes is manifest [42].

Our findings should also be considered in light of those from the ‘Primary care-led weight management for remission of type 2 diabetes trial’ (DiRECT) in which the remission of type 2 diabetes in response to an extended period of weight management through restriction of energy intake was studied [43] (note that DiRECT is a different study to IMI DIRECT). The results from a follow-up study to DiRECT, where a subgroup of participants (*n* = 88) underwent more detailed physiological testing similar to those in the present study, is of particular importance [44]. In this study neither liver fat nor pancreatic fat differed statistically between responders (those who remained free from type 2 diabetes after 12 months) and non-responders, though it did decrease in both groups in response to the intervention compared with the control arm. Moreover, first-phase insulin secretion improved in the responders whereas no difference was observed in the non-responders. The implication of this is that a reduction in both liver and pancreatic fat may coincide with remission of type 2 diabetes, and even be necessary, but it is not sufficient (as non-responders also had a decrease in both). The lack of a relationship between pancreatic fat and glucose sensitivity in our study could be for the same reason as in DiRECT.

A weakness of this analysis is that it is cross-sectional and, thus, the direction of the effects between some variables in the model cannot be easily ascertained. Nevertheless, the effects tested here and the structure of the models were prespecified, based on a biologically plausible hypothesis proposed previously [45]. As we have sought to test specifically the TC here we have also limited the model to edges (and directions) reflecting this hypothesis only. For this reason the model does not include some commonly hypothesised direct effect edges such as beta cell glucotoxicity and lipotoxicity (not mediated by pancreatic fat) [46], the rate sensitivity and potentiation fraction ratio variables in beta cell function modelled from oral glucose tolerance tests (often included alongside glucose sensitivity) [22].

Another consideration is the mathematical relatedness of fasting insulin, fasting glucose and 2 h glucose with insulin sensitivity, which are determined using some of the same variables derived from the fsOGTT/MMTT and are thus implicitly correlated. Nevertheless, the OGIS method has been extensively validated and the OGIS estimate has shown to be a close representation of the gold-standard *M* value from a euglycaemic–hyperinsulinaemic clamp [47]. As such, it is also worth bearing in mind that OGIS, as a whole-body insulin sensitivity measure, will not only reflect hepatic insulin sensitivity but also skeletal muscle insulin sensitivity. This is particularly important for the results of this study where the effects of physical activity on glycaemic control mediated through insulin sensitivity likely reflect an insulin-sensitising effect on skeletal muscle and not only the liver.

It is possible that the methods used here to normalise data distributions and control for confounding reduce statistical power to detect effects. Despite this caveat, the approach is valuable, as it renders the effects estimated within and between the two cohorts directly comparable, minimising confounding and bias, while also restricting the parameterisation of the model. An important limitation of any multivariable analysis, where ‘conditioning’ variables (covariates) are on the causal pathway between the defined exposures and outcomes, is that effects will be underestimated owing to mediation [30, 48]. In the current analysis, this is unlikely to be a cause for concern, as physical activity, for example, does not determine sex or chronological age; thus, neither sex nor age can mediate the effects of physical activity on metabolic outcomes.

### Conclusion

This analysis highlights that peripheral insulin sensitivity and liver fat are likely to be major mediators of the effects of physical activity in whole-body glucose homeostasis, key features of the pathogenic cycle proposed by Taylor [15, 16]. This study illustrates the value of large well-phenotyped cohorts, where emphasis is placed on detailed phenotypic assessments that allow structures of physiological relationships to be modelled and complex multi-dimensional pathogenic processes to be assessed.

## Electronic supplementary material

ESM Figure (PDF 129 kb)

## Data Availability

Requests for access to IMI DIRECT data, including data presented here, can made to DIRECTdataaccess@Dundee.ac.uk.

## Abbreviations

CFI: Comparative fit index
DiRECT: Primary care-led weight management for remission of type 2 diabetes trial
fsOGTT: Frequently sampled OGTT
hpfVM: High-pass filtered vector magnitude
IMI DIRECT: Innovative Medicines Initiative Diabetes Research on Patient Stratification
MMTT: Mixed-meal tolerance test
NAFLD: Non-alcoholic fatty liver disease
OGIS: Oral glucose insulin sensitivity
RMSEA: Root mean square error of approximation
TC: Twin-cycle hypothesis
TC-PA: Twin-cycle plus physical activity
TLI: Tucker–Lewis index
