# Supplementary material for: The role of physical activity in metabolic homeostasis before and after the onset of type 2 diabetes: an IMI DIRECT study
Source: Diabetologia. 2020 Jan 30;63(4):744–56. doi: 10.1007/s00125-019-05083-6 (PMC7054368; doi:10.1007/s00125-019-05083-6)
Supplement: Supplementary file 1 — (PDF 129 kb) [file 125_2019_5083_MOESM1_ESM.pdf]

ESM Fig. 1: Pairwise correlation matrix.

Koivula et al. The role of physical activity in metabolic homeostasis before and after the onset of type 2 diabetes: An IMI DIRECT study

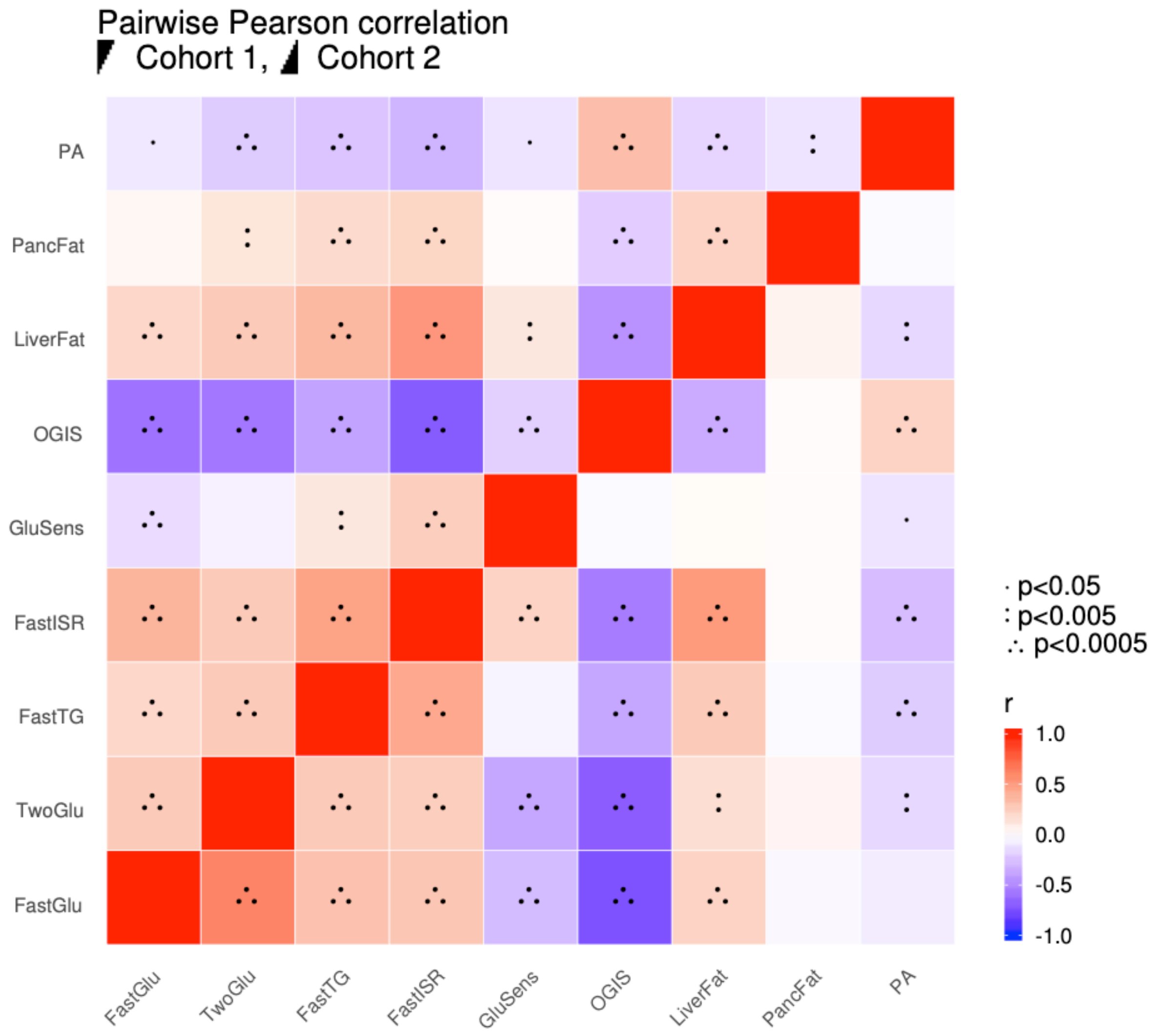

ESM Fig. 1 Pairwise correlation matrix. Fill color indicates Pearson correlation coefficient (r), where positive is denoted by red fill, inverse by blue fill, and magnitude by intensity. Cohort 1 and 2 are separate, above and below diagonal, respectively. All continuous variables were normally transformed and adjusted for age, sex, metformin treatment, study center, total energy-, carbohydrate-, fat-, and protein-intake. PA: Physical activity, FastGlu: Fasting glucose, TwoGlu: 2-hr glucose, OGIS: Oral glucose insulin sensitivity, LiverFat: Liver fat, PancFat: Pancreatic fat, FastISR: Fasting insulin secretion rate, GluSens: Glucose sensitivity (insulin secretion per glucose).
